# Mechanisms of Oxidative Stress and Therapeutic Targets following Intracerebral Hemorrhage

**DOI:** 10.1155/2021/8815441

**Published:** 2021-02-21

**Authors:** Zhenjia Yao, Qinqin Bai, Gaiqing Wang

**Affiliations:** ^1^Neurology, Shanxi Medical University, 030001 Taiyuan, Shanxi, China; ^2^Neurology, Sanya Central Hospital (Hainan Third People's Hospital), 572000 Sanya, Hainan, China

## Abstract

Oxidative stress (OS) is induced by the accumulation of reactive oxygen species (ROS) following intracerebral hemorrhage (ICH) and plays an important role in secondary brain injury caused by the inflammatory response, apoptosis, autophagy, and blood-brain barrier (BBB) disruption. This review summarizes the current state of knowledge regarding the pathogenic mechanisms of brain injury after ICH, markers for detecting OS, and therapeutic strategies that target OS to mitigate brain injury.

## 1. Introduction

ICH is a type of stroke characterized by spontaneous and nontraumatic bleeding in the brain that is associated with high morbidity and mortality rates [1]. ICH can be classified as primary and secondary. While treatment options for the former are limited, various strategies have been proposed for managing the latter [1].

Hematomal and perihematomal regions are biochemically active environments that sustain oxidative damage following ICH [2]. OS is defined as an imbalance between the formation of strong oxidants and physiologic antioxidant capacity [3]. ROS such as oxygen free radicals (e.g., superoxide (O_2_^−^) and hydroxyl radicals (OH^−^)) and nonradical compounds (e.g., hydrogen peroxide (H_2_O_2_) and hypochlorous acid), as well as reactive nitrogen species (RNS; e.g., nitric oxide (NO)) and a variety of nitrogenous compounds produced as metabolic byproducts, are the major drivers of oxidative damage [4] to proteins, lipids, and nucleic acids, which can induce inflammation, autophagy, apoptosis, and destruction of the BBB. OS is associated with dysregulation of cellular oxidation and reduction (redox) mechanisms; redox-sensitive thiols that are easily oxidized by nonradical oxidants such as H_2_O_2_ after ICH are essential for transcription factor regulation (e.g., nuclear factor erythroid 2-related factor (Nrf) 2 and nuclear factor- (NF-) *κ*B) [5].

The Kelch-like ECH-associated protein (Keap) 1/Nrf2/antioxidant response element (ARE) signaling pathway is the main regulatory system protecting cells against oxidative damage. Nrf2 is a master regulator of the cellular response to oxidative stress, which is associated with the expression of antioxidant and detoxification enzymes and factors such as NAD(P)H: quinone oxidoreductase (NQO) 1, catalase (CAT), superoxide dismutase (SOD), heme oxygenase- (HO-) 1, glutathione peroxidase (GPX), and glutathione-S-transferase (GST) [6]. Nrf2 was shown to mitigate early brain injury after ICH by translocating to the nucleus following activation and binding to AREs to activate the transcription of genes encoding antioxidant enzymes [7].

## 2. ROS Production after ICH

### 2.1. Production of ROS by Activated Phagocytes and Nonphagocytic Cells following ICH

Activated neutrophils, microglia, and macrophages are the main sources of ROS following ICH. Nicotinamide adenine dinucleotide phosphate (NADPH) oxidase (NOX) is expressed on the surface of neutrophils and macrophages and stimulates the production of ROS in response to extracellular signals such as hormones and cytokines [8]. Nonphagocytic cells such as neurons, microglia, astrocytes, and cerebrovascular endothelial cells also express NOX [8–10]. To date, 7 NOX isozymes have been identified in nonphagocytic cells that use NADH or NADPH as an electron donor for ROS production [11]. While NOX activity is generally low in these cells, they continuously produce O_2_^−^ even in the absence of external stimulation [11]. Hypoxia after ICH induces conformational changes in gp91phox, the heme-binding subunit of NOX, which activates the protein and leads to the formation of NOX complexes and increased ROS production [12] The activation of NOX was also reported to be the main mechanism underlying ROS generation in a rabbit model of intraventricular hemorrhage (IVH) [13], and OS resulting from NOX activation was shown to contribute to collagenase-induced ICH and brain injury [14]. NOX2 protein level was upregulated in the striatum of mice 12 h after ICH, which peaked at 24 h [15], and another study found that gp91phox was primarily expressed in activated microglia and colocalized with peroxynitrite (ONOO−) 24 h after ICH in the injured hemisphere [16]. However, following ICH, activated leukocytes release myeloperoxidase (MPO), which catalyzes lipid peroxidation and causes OS at the site of injury [17]. Additionally, increased expression of inducible NO synthase (iNOS) in M1 microglia in conjunction with the release of proinflammatory mediators and cytotoxic substances caused significant tissue damage after ICH [18].

### 2.2. Increased ROS Production in Mitochondria following ICH

Another important ROS is O_2_^−^ produced by mitochondria, which is generated as a byproduct of biological oxidation during mitochondrial respiration under physiologic conditions [19]. In most cells, the electron transport chain consumes 90% of cellular oxygen; 2% of this is transformed into oxygen free radicals in the mitochondrial inner membrane and matrix [20, 21]. Electrons that leak from the respiratory chain react with oxygen to form O_2_^−^. Mitochondrial O_2_^−^ is detoxified to H_2_O_2_ by SOD, then to O_2_ and H_2_O by antioxidant enzymes such as CAT and GPX. However, O_2_^−^ that elude antioxidant mechanisms can damage proteins, lipids, and DNA [21]. There are 7 known sources of O_2_^−^ in mammalian mitochondria: the ubiquinone-binding sites in complex I (site IQ) and complex III (site IIIQo), glycerol 3-phosphate dehydrogenase, complex I flavin (site IF), electron-transferring flavoprotein: Q oxidoreductase in fatty acid beta oxidation, pyruvate, and 2-oxoglutarate dehydrogenase, with site IQ and site IIIQo having the highest production capacities [21].

Mitochondria are storage sites for calcium ions (Ca^2+^). Under ischemia/reperfusion (I/R), excessive glutamate levels can cause an influx of Ca^2+^ into neurons via N-methyl-d-aspartic acid receptor (NMDAR), a ligand-gated ion channel [22]. Activation of the NMDAR leads to further Ca^2+^ influx, with increased levels in the cytosol and mitochondrial Ca^2+^ loading. Thrombin produced after ICH leads to Src kinase activation by activating protease-activated receptor 1 (PAR1), which phosphorylates and activates NMDAR. PARs are a subfamily of G protein-coupled receptors (GPCRs) with four members, namely, PAR1, PAR2, PAR3, and PAR4. PAR1 is highly expressed in many different cell types. PAR1 plays an important role in astrocyte proliferation, stimulus-induced long-term potentiation (LTP), and nerve growth factor (NGF) secretion. PAR1 enhances Src-mediated tyrosine phosphorylation of NMDA receptor in ICH [23]. Activation of *α*-amino-3-hydroxy-5-methyl-4-isoxazolepropionic acid (AMPA) receptor by glutamate after ICH in motor neurons also increased Ca^2+^ and Na+ influx and mitochondrial Ca^2+^ loading [22]. Following ICH, Ca^2+^ stored in the endoplasmic reticulum (ER) is thought to be sequestered by mitochondria. Mitochondrial Ca^2+^ loading reduces mitochondrial membrane potential (MMP) and opens the mitochondrial permeability transition pore (MPTP), resulting in mitochondrial damage and disruption of the mitochondrial respiratory chain; together, these processes result in the release of excess ROS [22].

### 2.3. Increased ROS Production by the ER following ICH

The ER is the site of protein synthesis, posttranslational modification, folding, and trafficking. ICH can cause ER stress (ERS), which is characterized by protein misfolding, accumulation of abnormal proteins, and Ca^2+^ imbalance, all of which trigger the unfolded protein response (UPR) [24]. Glutamate excitotoxicity and the inflammatory response can result in ERS. When ICH causes ERS, ROS are generated by NOX4 in the internal membrane. ROS then acts as a signaling intermediate that subsequently mitigates ERS via the UPR. If ERS is not alleviated, the delayed expression of proteins such as C/EBP homologous protein (CHOP) causes a secondary increase in ROS levels [25]. Additionally, disulfide bonds in proteins translated in the ER are highly sensitive to changes in redox balance; thus, both reducing and oxidizing conditions can disrupt protein folding and cause ERS. On the other hand, oxidative protein folding is a major source of intracellular ROS production [26]; during this process, thiol groups on the cysteines of peptides are oxidized and form disulfide bonds [26]. After accepting electrons from protein disulfide isomerase (PDI), ER oxidoreductin (ERO) 1 transfers electrons to molecular oxygen to generate H_2_O_2_, the major type of ROS formed in the ER lumen [26]. In the ERS following ICH, disruption of disulfide bond formation leads to ROS accumulation and OS [26]. Inositol 1,4,5-trisphosphate receptor and voltage-dependent anion channel—which are located in the ER and mitochondria, respectively—form a complex with the chaperone protein glucose-regulated protein (GRP) 75, thus physically connecting the 2 organelles [22]. Upon ERS, Ca^2+^ transfer at the contact points between the ER and mitochondria leads to mitochondrial dysfunction, thereby increasing mitochondrial ROS production, resulting in cellular stress or neuronal death. Although there have been few studies investigating the relationship between ERS and ICH, given the interaction between ERS and microglial activation [27], neuroinflammation, and autophagy after ICH, clarification of this point can inspire new avenues for ICH treatment.

### 2.4. Hemoglobin (Hb) Toxicity after ICH

Hb toxicity is induced by free radicals generated via Fenton-type reactions and by oxidative damage to proteins, nucleic acids, and lipids [28]. During its conversion to methemoglobin, oxyhemoglobin releases O_2_^−^, which in turn forms OH− and contributes to ROS production [29]. Hb, a major component of erythrocytes, is a heterotetramer composed of *α* and *β* globin subunits that each bind a heme molecule. Hb induces the expression of iNOS by M1 microglia and neutrophils after ICH. NOS is expressed by endothelial cells, macrophages, neurophagocytes, and nerve cells; there are 2 isoenzymes besides iNOS—namely, neural and endothelial NOS [30]. Overexpression of iNOS or endothelial NOS and the consequent overproduction of NO lead to changes in tight junction proteins and can potentially disrupt the BBB [31]. Following ICH, heme is released by Hb and decomposed into bilirubin, free iron, and carbon monoxide.

### 2.5. Increased ROS Production by Heme following ICH

Heme (ferrous protoporphyrin IX) is a reactive, low molecular weight form of iron that participates in Fenton-type oxygen radical reactions in neurons, microglia, and neutrophils [32]. Hemin, the oxidized form of heme, accumulates in intracranial hematomas and is a potent oxidant [33]. Hemin is bound by hemopexin in serum, and the complex is translocated into the cell via lipoprotein receptor-related protein (LRP) 1. Intracellular hemin is degraded into bilirubin, Fe^2+^, and carbon monoxide. Fe^2+^ derived from hemin can generate OH−—the most reactive oxygen radical—via the Fenton reaction, leading to an increase in ROS levels [22].

### 2.6. Increased ROS Production from Ferrous Iron and Ferritin following ICH

**Figure 1:**
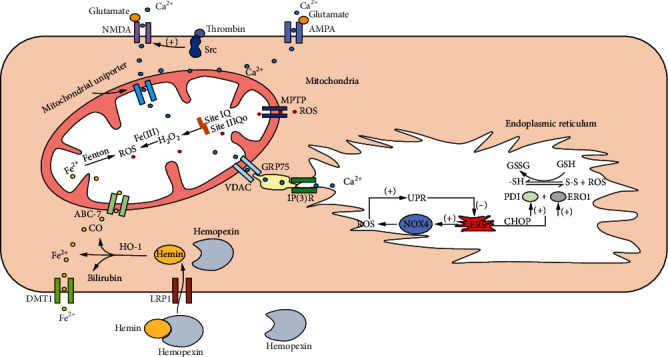
The production of reactive oxygen species after cerebral hemorrhage: hemin and divalent iron ions enter the cell through the corresponding receptors on the cell membrane, and further, Fenton reaction occurs in the mitochondria, thereby generating excess ROS; glutamate and thrombin activate ion channel receptors on the cell membrane to promote Ca^2+^ influx; in addition, the VDAC and IP(3)R located in the mitochondria and the endoplasmic reticulum are connected through GRP75, so that the Ca^2+^ in the endoplasmic reticulum flows into the mitochondria, and the Ca^2+^ in the mitochondria is further overloaded, and the MPTPA channel on the mitochondria is opened and released. The H_2_O_2_ produced at the two sites of site IQ and site IIIQo in the mitochondria generates ROS when it encounters Fe(III). Endoplasmic reticulum UPR can reduce ERS. When ERS cannot be relieved, certain UPR components (such as C/EBP homologous protein CHOP) may cause oxidative stress; in addition, in the stressed ER, the imbalance of disulfide bond formation and breaking may lead to the accumulation of reactive oxygen species (ROS) and cause oxidative stress. ROS: reactive oxygen species; Fe^2+^: ferrous iron; AMPA: *α*-amino-3-hydroxy-5-methyl-4-isoxazole-propionic acid receptor; NMDA: N-methyl-D-aspartic acid receptor; HO-1: heme oxygenase-1; DMT: divalent metal transporter 1; MPTP: mitochondrial permeability transition pore; VDAC: voltage-dependent anion channel; ER: endoplasmic reticulum; IP3R: inositol 1,4,5-trisphosphate receptor; ERO1: ER oxidase 1; CHOP: C/EBP homologous protein; UPR: unfolded protein response; PDI: protein disulfide isomerases; LRP1: lipoprotein receptor-related protein; NOX4: adenine dinucleotide phosphate oxidase 4.

Ferrous iron is one of the main contributors to OS following ICH. Free iron catalyzes the conversion of O_2_^−^ and H_2_O_2_ into OH− via the Fenton reaction while oxidizing iron from a divalent to a trivalent form [34]. Ferrous iron is transported into the cell through divalent metal transporter (DMT) 1 and into mitochondria by ATP-binding cassette- (ABC-) 7 [22], resulting in OS. Ferritin functions as a source of iron in lipid peroxidation; the release of iron from ferritin is mediated by O_2_^−^ [34] (Figure 1). Knowledge of the mechanisms and dynamics of ROS generation following ICH can guide the development of drugs for the treatment of ICH that act by mitigating OS.

## 3. OS-Induced Brain Damage following ICH

### 3.1. Organelle Damage by OS

Oxidative damage in DNA includes base modifications and hydrogen bond breakage [35]; in proteins, it can include amino acid modifications, peptide chain fractures, and protein cross-linking [36]. Free radicals cause cellular membrane damage by promoting lipid peroxidation (especially of polyunsaturated fatty acids); the binding of free radicals to membrane receptors also leads to the destruction of membrane integrity [37].

### 3.2. OS-Induced Autophagy in ICH

Autophagy is a cellular process for the clearance of damaged organelles and proteins that are misfolded or no longer required. Autophagy is triggered by ICH-induced OS [38]. Studies have indicated that superoxide is the major form of ROS regulating autophagy [39]. A hallmark of the process is the reversible conjugation of autophagy-related protein (Atg) 8 to the autophagosome membrane. Under conditions of serum starvation, ROS (especially H_2_O_2_) induces the inactivation of Atg4 during autophagosome formation, which promotes the lipidation of Atg8. As the autophagosome matures and fuses to a lysosome, the reduction in H_2_O_2_ levels promotes the activation of Atg4 along with the delipidation and recycling of Atg8 [39]. The regulation of autophagy is closely related to p62/Keap1/Nrf2 redox signaling. The autophagy-related factor p62 binds to ubiquitinated protein aggregates, and its affinity for Keap1 is increased upon phosphorylation at Ser351 [40]. This induces the degradation of Keap1 via autophagy and releases Nrf2, which accumulates and translocates to the nucleus where it binds to AREs to activate the transcription of genes encoding antioxidant enzymes and p62 as well as other autophagy-related factors [40]. ROS produced as a result of ICH act on the mammalian target of rapamycin complex (mTORC) 1/UNC51-like kinase (ULK) 1 and AMP-activated protein kinase (AMPK)/ULK1 signaling pathways that regulate autophagy. AMPK activation and mTORC1 inhibition in response to ROS lead to ULK1 activation and induction of autophagy [40]. Activated ULK1 phosphorylates its interaction partners Atg13 and FAK family-interacting protein of 200 kDa (FIP200), resulting in the activation of the class III phosphoinositide 3-kinase (PI3K) complex via activating molecule in BECN1-regulated autophagy (AMBRA) 1, thus initiating the nucleation of autophagosomes from the ER or mitochondria [40]. Some research data suggest that oxidative stress induces autophagy activation, which may make ICH-induced brain injury disappear [41]. This may also reduce early brain damage in SAH [42]. However, others believe that under- or overactivation of autophagy may lead to cell damage and death. The potential role of selective autophagy in the clinical treatment of hemorrhagic stroke has been recognized. The mechanism of autophagy activation mediated by mitochondrial and ERS induced by OS after ICH remains to be studied.

### 3.3. Apoptosis Induced by OS following ICH

Severe OS and a high intracellular concentration of Ca^2+^ following ICH induces MPTP opening and a reduction in MMP, with subsequent release of cytochrome C and other proapoptotic proteins from the mitochondrial membrane into the cytosol, which activates the intrinsic neuronal apoptosis pathway in mitochondria [43]. After cerebral hemorrhage (ICH), oxidative stress leads to DNA and protein damage, which leads to neuronal apoptosis.

### 3.4. Matrix Metalloproteinase (MMP) Activation by OS after ICH

Following ICH, O_2_^−^ and NO levels are increased as a result of endothelial NOS and iNOS activities, respectively. In the context of cerebral I/R, NO reacts with O_2_^−^ produced by gp91phox to form ONOO−. The activation of MMP-9 and MMP-2 by ONOO− results in the degradation of the tight junction proteins claudin-5 and occludin as well as the extracellular matrix, leading to disruption of the BBB [44]. ONOO− induces other forms of cellular damage including protein oxidation, DNA damage, lipid peroxidation, tyrosine nitration, and mitochondrial dysfunction. Notably, tyrosine nitration leads to the modification of functional proteins and vascular endothelial cell injury [45]. Following ICH, Hb-induced OS resulting from MMP activation disrupted the BBB and induced cell apoptosis, which was reversed by overexpression of SOD1 [29].

### 3.5. OS Mediates the Inflammatory Response following ICH

**Figure 2:**
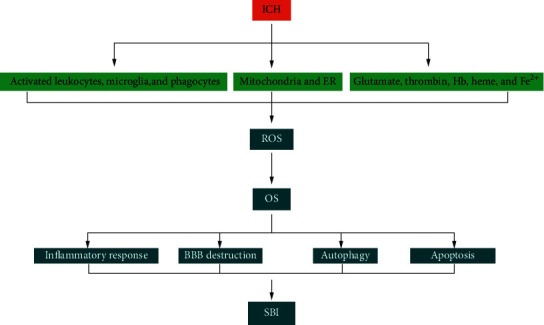
Summary of mechanisms by which OS aggravates SBI after ICH: after ICH, activated phagocytes, mitochondria, ER, and RBC lysates all cause excess release of ROS. This increase in OS exacerbates the inflammatory response, apoptosis, autophagy, and BBB disruption, with further SBI aggravation. BBB: blood-brain barrier; ICH: intracerebral hemorrhage; OS: oxidative stress; ROS: reactive oxygen species; SBI: secondary brain injury.

ROS-induced activation of the NACHT, LRR, and PYD domain-containing protein (NLRP) 3 inflammasome following ICH results in the release of interleukin- (IL-) 1*β*, which promotes neutrophil infiltration, inflammation, and brain edema [46]. ROS and RNS produced by neutrophils regulate the inflammatory response after ICH by modulating phagocytosis, cellular function, gene expression, and apoptosis [47]. In the context of ICH, MPO from activated white blood cells catalyzes the oxidation of chloride ions by H_2_O_2_, producing the strong oxidant hypochlorous acid (HOCl), which can cause further tissue damage and promote inflammation [48]. Thiol redox circuits are a normal part of cell signaling and physiologic regulation; their destruction in vascular disease causes OS, leading to the activation of a proinflammatory signaling cascade [5]. The net effect of NOX activation may be proinflammatory, as evidenced by its activation in phagocytes after ICH and the resultant generation of reactive oxygen intermediates that enhance inflammation and tissue damage [8] and further increase OS. The infiltration of inflammatory cells induces an influx of Ca^2+^, increasing free radical production, and lipid peroxidation. After ICH, M1 phenotypic microglia can be activated by thrombin to release proinflammatory cytokines and chemokines such as interleukin-1*β* (IL-1*β*), tumor necrosis factor-*α* (TNF-*α*), and ROS, thereby attracting surrounding inflammatory mediators. In addition, microglia strongly express HO-1, which converts heme into iron, carbon monoxide (CO), and biliverdin [45] (Figure 2).

## 4. Markers for Detecting OS after ICH

### 4.1. Oxidative DNA Damage

#### 4.1.1. 8-Oxo-7,8-Dihydro-2′Deoxyguanosine (8-OHdG) and 8-Oxo-7,8-Dihydro-Guanine (8-oxoGua)

8-OHdG and 8-oxoGua are sensitive markers for DNA oxidative damage that are produced through hydroxylation at the C-8 position of guanine. Various methods are used to measure 8-oxoGua and 8-oxodG concentrations in blood and urine samples, including gas chromatography-mass spectrometry (GC-MS), high-performance liquid chromatography-electrochemical detection (HPLC-ECD), immunohistochemistry, and enzyme-linked immunosorbent assay (ELISA). GC-MS and HPLC-ECD have higher specificity and are more accurate than ELISA [49]. At 24 h after ICH, 8-OHdG was detected at high levels at the borders of damaged and normal tissues, coinciding with an increase in the number of apurinic/apyrimidinic sites; expression peaked in the first 3 days post-ICH and returned to baseline starting from day 7 [50]. The OS marker leukocyte 8-hydroxy-2′-deoxyguanosine was shown to be an independent predictor of 30-day outcome following ICH [51], while serum 8-OHdG and 8-oxoGua levels were related to 30-day mortality in ICH patients [52]. According to reports, the optimal cutoff of serum OGS levels in ICH patients is 4.94 ng/mL (according to Youden's J index). When the OGS level of patients is higher than the cutoff value, the mortality rate is higher [52].

### 4.2. Lipid Peroxidation

#### 4.2.1. Malondialdehyde (MDA)

ICH leads to progressively higher levels of lipid peroxidation, as evidenced by increased diene conjugation and MDA levels [52]. MDA is the most commonly used biomarker of lipid peroxidation in clinical studies. Serum MDA levels were shown to increase rapidly at the early stage of ICH and were closely related to the severity of clinical symptoms [53]. MDA can be accurately detected by HPLC [53], whereas the detection of thiobarbituric acid-reactive substances by ultraviolet light (UV) spectrophotometry overestimated MDA levels in human plasma [43]. Serum MDA level at diagnosis of severe spontaneous ICH was shown to be associated with early mortality [54]. The study found that patients with spontaneous cerebral hemorrhage have a higher risk of death when the serum MDA is higher than 2.48 nmol/mL [54]. It is common to use the thiobarbituric acid reactive substance (TBARS) method to determine the serum MDA of cerebral hemorrhage mouse models and patients with ICH [2, 52, 54–58].

#### 4.2.2. 4-Hydroxynonenal (4-HNE)

4-HNE is a product of lipid peroxidation in the cell membrane that can be quantified by HPLC, GC-MS, LC-MS, and aldehyde-reactive probes [59]. 4-HNE levels in cerebrospinal fluid (CSF) and plasma samples of patients with Parkinson's disease have also been determined by GC-negative-ion chemical ionization mass spectrometry-based detection of O-pentafluorobenzyl oxime [60]. Vasospasm in patients with SAH has been evaluated based on measurement of lipid peroxidation; the levels of polyunsaturated fatty acid cyclization products, F2-isoprostanes (F2-IsoPs), and neuroprostanes were highest on day 1 post-SAH and decreased over time, with similar trends observed in the levels of 4-HNE, 4-oxononenal, and MDA [61].

#### 4.2.3. F2-IsoPs

Another hallmark of lipid peroxidation is the production of F2-IsoPs, which can be detected by LC-MS and the gold standard method GC-MS, while ELISA is less effective [62]. 8-Iso-prostaglandin (8-iso-PG) F2*α* is an isomer derivative of F2-IsoP that serves as a biomarker for evaluating OS and lipid peroxidation. 8-Iso-PGF2*α* is present in a free form in tissue or in esterified form in lipids; levels in the blood were shown to be increased after ICH, which was positively correlated with the National Institute of Health Stroke Scale score and hematoma volume [60], and elevated plasma concentrations of 8-Iso-PGF2*α* were reported to be associated with the clinical severity and outcome of ICH. The plasma level of 8-iso-PGF2*α* is an independent prognostic factor in ICH [63] and indicator of oxidation in analyses of F2-IsoP, isofuran, and F(4)-neuroprostane concentrations following aneurysmal SAH and traumatic brain injury. A higher level of F(4)-neuroprostanes in CSF more accurately reflects neural dysfunction than the elevated F2-IsoP level [64].

### 4.3. Enzyme Activity

#### 4.3.1. SOD

Studies have determined that the level of SOD in patients with cerebral hemorrhage (113.62 ± 9.14 U/mL) is significantly lower than healthy (161.20 ± 21.12 U/mL) [65]. It is a common indicator for evaluating oxidative stress in mouse models of ICH [2, 6, 56, 58, 66, 67].

#### 4.3.2. MPO

The activity of MPO, a potent oxidizing enzyme, can be determined by quantifying the level of 3-Cl-Tyr and the conversion of hydroethidine to 2-chloroethidium. The MPO/H_2_O_2_/chloride system of leukocyte activation is responsible for the generation of 3-Cl-Tyr, which is a biomarker of neuroinflammation [68]. Moreover, serum MPO concentration was found to be increased in ICH patients, which was correlated with ICH severity and prognosis [69]. In ICH animal experiments, MPO can be measured with immunofluorescence assay [55].

### 4.4. Evaluation of Antioxidant Levels

#### 4.4.1. Glutathione (GSH) and Oxidized Glutathione (GSSG)

The molar ratio of GSH to GSSG is a useful index of OS in ICH. Total GSH in cells is quantified by measuring the concentration of the glutathione-N-ethylmaleimide conjugate by UV/visible HPLC. However, under pathologic conditions, total GSH content is lower than that of GSSG. Spectrophotometry can be used to determine GSSG concentration by either the GSH recycling method or HPLC [70], while commercial GSH/GSSG detection kits are commonly used in studies involving ICH models [6, 57, 66].

#### 4.4.2. Allantoin

Allantoin is a physiologic antioxidant that can be detected in human plasma and serum samples by GC-MS [71]. It was also demonstrated that determination of urinary allantoin concentrations by GC-MS was useful for evaluating the efficacy of clinical interventions in preterm neonates diagnosed with germinal matrix IVH [64].

#### 4.4.3. Thioredoxin (TRX)

TRX is an antioxidant that eliminates oxygen as well as OH− radicals. Serum concentrations of TRX can be determined by ELISA, with commercial kits widely available. Increased serum concentrations of TRX were shown to be related to hemorrhage severity and long-term mortality in patients with ICH [72].

Besides, the mouse ICH model can also use the ROS analysis kit to detect the level of oxidative stress. This kit uses the principle of the fluorescent probe 2′,7′-dichlorofluorescein diacetate to determine [6, 57, 67, 73].

## 5. Therapeutic Strategies Targeting OS following ICH (Supplementary Table 1–2)

### 5.1. Regulation of Oxidant Signaling Pathways

#### 5.1.1. Keap1/Nrf2/ARE Signaling (Supplementary Table 1)

**Figure 3:**
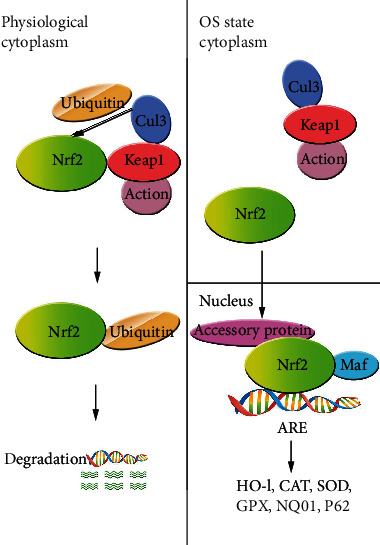
Description of antioxidant enzyme system regulation via the Keap1-Nrf2-ARE pathway. (a) Under normal basal conditions, Keap1 binds Nrf2 and keeps its level low by ubiquitination and proteasomal degradation; (b) under OS conditions, Keap1 is oxidized by OS, and dissociation of Nrf2 from Keap1 enables Nrf2 to translocate to the nucleus. Nrf2 combines with the small Maf protein to form a Nrf2-Maf heterodimer, and Nrf2 binds to accessory protein and then ARE activates gene expression of HO-1, NQO1, GPX, SOD, CAT, and the autophagy protein p62. ARE: antioxidant response element; CAT: catalase; Cul3: Cullin3; GPX: glutathione peroxidase; HO-1: heme oxygenase-1; ICH: intracerebral hemorrhage; Keap1: Kelch-like ECH-associated protein 1; Maf: musculoaponeurotic fibrosarcoma; NQO1: NAPDH quinone oxidoreductase 1; Nrf2: nuclear factor erythroid 2-related factor 2; OS: oxidative stress; SOD: superoxide dismutase.

The Keap1/Nrf2/ARE signaling pathway is one of the most important defense mechanisms against OS in ICH [74] as it promotes the expression of endogenous antioxidant enzymes including NQO1, CAT, SOD, HO-1, and GPX [75]. Nrf2 was upregulated following ICH, with peak level occurring at 24 h; the time course of expression was shown to be correlated with the severity of brain edema and neurologic deficits. Heme oxygenase-1 is resistant to OS in the early stages of ICH but is thought to promote oxidation in subsequent stages of the disease process. Drugs targeting the Nrf2/ARE signaling pathway have therapeutic potential for reducing brain damage caused by OS and inflammation following ICH (Figure 3). The drugs used to treat ICH animal models by regulating the Nrf2-ARE signaling pathway include glycyrrhizin [6], simvastatin [76], methyl hydrogen fumarate [77], nicotinamide mononucleotide [56], astaxanthin [55], mangiferin [71], RS9 [78], silymarin [79], sulforaphane [80], Hb pretreatment [81], melatonin [82], and recombinant human erythropoietin [83], calycosin [84], (-)-epicatechin [67], luteolin [85], and ghrelin [86].

#### 5.1.2. Peroxisome Proliferator-Activated Receptor (PPAR) *γ* Signaling

PPAR*γ* regulates CAT expression and is another antioxidant signaling pathway. CAT is ubiquitously expressed in all cell types including glia and neurons and is predominantly localized in peroxisomes. A PPAR response element is present in the CAT gene promoter, indicating a direct regulatory interaction. 15-Deoxy-*Δ*12,14-prostaglandin J2 (15d-PGJ2) is a nonenzymatic breakdown product of prostaglandin D2; unlike synthetic thiazolidinediones, 15d-PGJ2 acts as an endogenous ligand for PPAR*γ* to promote the expression of CAT, which was shown to be associated with decreased inflammation, oxidative damage, and neuronal loss in a rat model of ICH [87]. It was noted in the cerebral hemorrhage model that telmisartan can induce the expression of receptor *γ* activated by endothelial nitric oxide synthase and peroxisome proliferators and reduce oxidative stress, apoptosis signals, and tumor necrosis factor-*α* and cyclooxygenase-2 expression [88].

### 5.2. Decreased ROS Production following ICH

#### 5.2.1. NOX

The inhibition of NOX reduces the generation of endogenous ROS. A small ubiquitin-related modifier was shown to negatively regulate NOX5 in human neutrophils and vascular smooth muscle cells, thus limiting the production of ROS. However, there is little known about the involvement of small ubiquitin-related modifiers in ICH [89]. Melatonin was previously found to inhibit ROS generation and OS after ICH [73]. Meanwhile, overexpression of the ubiquitin ligase ring finger (RNF) 34 exacerbated brain injury after ICH by promoting peroxisome proliferator-activated receptor coactivator- (PGC-) 1*α* degradation while stimulating the generation of mitochondrial ROS. Thus, genetic ablation of RNF34 is a potential strategy for the treatment of ICH [90]. The NADPH oxidase inhibitor apocynin improved the therapeutic efficacy of mesenchymal stem cells in the acute stage of ICH by exerting neuroprotective effects and enhancing the integrity of cerebral vasculature [12].

#### 5.2.2. Mitochondria

Sodium benzoate was reported to mitigate OS-induced secondary brain injury, inhibit neuronal apoptosis, and suppress ROS production in mitochondria after ICH via DJ-1/protein kinase B (AKT)/I*κ*B kinase (IKK)/NF-*κ*B signaling [91]. The alleviation of neurologic deficits by deferoxamine via inhibition of PGC-1*α* signaling was shown to reduce OS caused by mitochondrial dysfunction [92]. Besides, drugs that can reduce the oxidative stress induced by mitochondrial function damage in ICH animal models include Dexmedetomidine (Dex) [92], Pyrroloquinoline Quinone (PQQ) [93], and melatonin [73].

### 5.3. Elimination of ROS following ICH

Intracerebroventricular injection of recombinant *α*1-microglobulin (A1M) resulted in its coexistence with extracellular Hb, while injection of human A1M mitigated the inflammatory response and mitochondrial damage in a rabbit model of IVH [94]. As a radical scavenger, A1M eliminates both heme and radicals, thus providing early protection to the immature brain in preterm IVH [94]. The novel free radical scavenger NSP-116 was found to alter cerebral blood flow and alleviate neurologic deficits in a model of I/R injury caused by middle cerebral artery occlusion; it also suppressed the expansion of hematomas and reduced neurologic deficits [95]. Edaravone, a free radical scavenger, reduced cerebral edema, and lipid peroxidation following IVH in rats and repeated administration improved learning and memory performance [96]. Hydrogen gas was found to reduce OS damage by eliminating OH− in a rat model of cerebral I/R [97], and inhaled hydrogen diminishes OS and brain edema 24 h after ICH, although it did not improve clinical outcome [98]. In addition, Glibenclamide (GLI) [99] and tempol [100] are also free radical scavengers for oxidative stress after ICH.

### 5.4. Effects of Antioxidants following ICH

Melatonin (N-acetyl-5-methoxytryptamine) is an indolamine that is primarily synthesized by the pineal gland and can easily pass through the BBB. Melatonin improved severe ICH-induced brain injury by mitigating OS, apoptosis, inflammation, DNA damage, brain edema, and BBB damage and by inhibiting MPTP opening [73], and in a SAH model, it mitigated cerebral OS by increasing the expression of HO-1, NQO1, NADPH, and GST-*α*1, possibly via activation of the Nrf2/ARE signaling pathway [82]. Baicalein treatment reduced OS in rats by increasing the activity of SOD and GPX while downregulating the expression of MDA in the brain. Thus, baicalein can potentially be used to treat ICH and related brain injuries [101]. Danhong—a traditional Chinese medicine extracted from 2 herbs (Salviae miltiorrhiza Bunge (Danshen, China) and Carthamus tinctorius L (Honghua, China))—contains flavonoids and phenolic compounds and was reported to increase the expression of peroxiredoxin (Prx) 1 in astrocytes, thereby preventing severe brain injury following ICH in aged rats [102]. In addition, carnosine [103], COA-Cl (a novel synthesized nucleoside analog) [104], AE1-259-01 EP2 receptor agonists [105], green tea and red tea [57], protocatechuic acid (PCA) [106], nebivolol [107], 14Adiponectin (APN) [108], metformin [109], C1q/tumor necrosis factor-related protein 3 (CTRPs) [15], gastrodin [110], Naringin (NGN) [111], and Parthenolide (PN) [112] are all effective antioxidants in ICH animal models.

## 6. Summary and Outlook

The pathophysiologic processes that occur after ICH are complex, involving the neuroinflammatory response, OS, cytotoxicity caused by erythrocyte lysis, and the production of thrombin. Elucidating the causes of brain injury and the underlying molecular mechanisms and identifying novel markers of OS in the context of ICH will enable the development of effective interventions for the prevention and treatment of secondary brain injury following ICH.

## Data Availability

The data that support the findings of this study are openly available in PubMed at https://pubmed.ncbi.nlm.nih.gov/. Data sharing is not applicable to this article as no new data were created or analyzed in this study.

## Supplementary Materials

Supplementary Materials Table 1: potential Nrf2 agonists against oxidative stress in ICH. Table 2: the potential therapy targets at the oxidative stress caused by ICH.
