# Supplementary material for: Mechanisms of Oxidative Stress and Therapeutic Targets following Intracerebral Hemorrhage
Source: Oxid Med Cell Longev. 2021 Feb 21;2021:8815441. doi: 10.1155/2021/8815441 (PMC7920740; doi:10.1155/2021/8815441)
Supplement: Supplementary Materials — Table 1: potential Nrf2 agonists against oxidative stress in ICH. Table 2: the potential therapy targets at the oxidative stress caused by ICH. [file 8815441.f1.docx]

**Supplementary tables**

**Table1 Potential Nrf2 agonists against Oxidative Stress in ICH**

| Drugs | Measures |
| --- | --- |
| Glycyrrhizin | 20mg/kg ( intra-peritonelly, ip) by mitigating Blood-brain barrier permeability and antioxidating in ICH rats[6] |
| Simvastatin | 2mg/kg ( ip) by decreasing SOD,GSH,GSSG,MDA and NO in ICH rats [76] |
| Nicotinamide mononucleotide (NMN) | 300mg/kg (Tail vein injection) by decreasing MDA and H 2 O 2 in ICH mice [56] |
| Astaxanthin(ATX) | 20μL (Intraventricular injection) by antioxidating in SAH rats [55] |
| Mangiferin (MF) | 100mg/kg (ip) by antioxidating in SAH rats [71] |
| Silymarin | 200mg/kg(ip) by decreasing ROS production in ICH rats[79] |
| Sulforaphane (SUL） | 5mg/kg (ip) by promoting antioxidation in SAH rats[80] |
| Melatonin | 150mg/kg (ip) by promoting antioxidation in SAH rats[82] |
| Recombinant human erythropoietin (rhEPO) | 1000U/kg (ip) by promoting antioxidation in SAH rats [83] |
| Luteolin | 10mg/kg (ip) by promoting antioxidation and HO-1 in ICH rats [85] |
| Ghrelin | 20 μg (ip) by antioxidating in ICH rats[86] |

**Table 2. The potential therapy targets at the oxidative stress caused by ICH**

| **Mechanisms** | **Potential Therapy Targets** |
| --- | --- |
| Reducing mitochondrial dysfunction mediated by  oxidative stress | Melatonin (5 mg/kg, intra-peritonelly, ip) in ICH rats [73] |
|  | Pyrroloquinoline Quinone (10 mg/kg, intragastrical,ig) in ICH rats[93] |
|  | Dexmedetomidine( 25 µg/kg/day,ip) in ICH mice [92] |
|  | Sodium Benzoate ( 200 mg/kg, ip) in ICH rats [91] |
| Free radical scavenger | α1-microglobulin(rA1M 25μl,9.4mg/ml, Intraventricular injection, ICV) in IVH rabbits[94] |
|  | Tempol (100 mg/kg, Intravenous injection,iv) in ICH rats [100] |
|  | NSP-116(30 mg/kg/day, ig) in MCAO rats [95] |
|  | Glibenclamide (10 µg/kg, 30 min pre-operation, then 1µg/kg/day, ip) in ICH rats [99] |
|  | Edaravone ( 6mg/kg, Subcutaneously) in ICH rats[96] |
| Antioxidants | Carnosine (1g/kg, ip)  in ICH rats [103] |
|  | Baicalein (50mg/kg, ip)in ICH rats [101] |
|  | COA-Cl (30 µg / kg, ip) in ICH rats[104] |
|  | Green Tea and Red Tea(400mg/ml/day, ig) in ICH rats[57] |
|  | Protocatechuic acid(50mg/kg, ip ) in ICH mice[106] |
|  | Nebivolol (10mg/kg, ig ) in SAH rats[107] |
|  | Adiponectin(15mg/kg, ip) in ICH rats[108] |
|  | Metformin (100 mg/kg, ig) in ICH rats [109] |
|  | Gastrodin(2ml,100mg/kg,ip) in ICH rats[110] |
|  | Naringin (40 mg/kg,ig ) in ICH rats [111] |
|  | Parthenolide(1 mg/kg, ip) in ICH rats[112] |
